# Stable Reference Gene Selection for RT-qPCR Analysis in Nonviruliferous and Viruliferous *Frankliniella occidentalis*

**DOI:** 10.1371/journal.pone.0135207

**Published:** 2015-08-05

**Authors:** Chunxiao Yang, Hui Li, Huipeng Pan, Yabin Ma, Deyong Zhang, Yong Liu, Zhanhong Zhang, Changying Zheng, Dong Chu

**Affiliations:** 1 Hunan Academy of Agricultural Sciences, Institute of Plant Protection, Changsha, Hunan, China; 2 College of Agronomy and Plant Protection, Qingdao Agricultural University, Qingdao, China; 3 Department of Entomology, University of Kentucky, Lexington, Kentucky, United States of America; 4 Hunan Academy of Agricultural Sciences, Hunan Vegetable Institute, Changsha, Hunan, China; Institute of Vegetables and Flowers, Chinese Academy of Agricultural Science, CHINA

## Abstract

Reverse transcriptase-quantitative polymerase chain reaction (RT-qPCR) is a reliable technique for measuring and evaluating gene expression during variable biological processes. To facilitate gene expression studies, normalization of genes of interest relative to stable reference genes is crucial. The western flower thrips *Frankliniella occidentalis* (Pergande) (Thysanoptera: Thripidae), the main vector of *tomato spotted wilt virus* (TSWV), is a destructive invasive species. In this study, the expression profiles of 11 candidate reference genes from nonviruliferous and viruliferous *F*. *occidentalis* were investigated. Five distinct algorithms, *geNorm*, *NormFinder*, *BestKeeper*, the *ΔC*_*t*_ method, and *RefFinder*, were used to determine the performance of these genes. *geNorm*, *NormFinder*, *BestKeeper*, and *RefFinder* identified *heat shock protein 70* (*HSP70*), *heat shock protein 60* (*HSP60*), *elongation factor 1 α*, and *ribosomal protein l32* (*RPL32*) as the most stable reference genes, and the *ΔC*_*t*_ method identified *HSP60*, *HSP70*, *RPL32*, and *heat shock protein 90* as the most stable reference genes. Additionally, two reference genes were sufficient for reliable normalization in nonviruliferous and viruliferous *F*. *occidentalis*. This work provides a foundation for investigating the molecular mechanisms of TSWV and *F*. *occidentalis* interactions.

## Introduction

The western flower thrips, *Frankliniella occidentalis* (Pergande) (Thysanoptera: Thripidae), is a destructive species that is found globally. This insect damages hundreds of plant species through direct and indirect mechanisms via feeding and transmitting tospoviruses, respectively [1–3]. The *tomato spotted wilt virus* (Family: Bunyaviridae; Genus: Tospovirus; TSWV) is transmitted in a circulative, propagative manner by thrips, with *F*. *occidentalis* as the most efficient vector of TSWV [4]. Currently, TSWV ranks among the top ten most economically important plant viruses worldwide [5]. TSWV infects in as many as 1,000 plant species, causing disease in many vegetables and ornamentals [6]. Global epidemics of TSWV are likely related to the worldwide distribution of *F*. *occidentalis* [7].

To better understand the molecular mechanisms of TSWV and *F*. *occidentalis* interactions, the transcriptomes of *F*. *occidentalis* were generated [8–11]. Additionally, RNA interference (RNAi) tool that enables functional genomics assays was successfully developed for *F*. *occidentalis* [12]. Given the nature of RNAi mechanisms, the impact of RNAi will likely be a method to control gene expression profiles of pest organisms. To make better use of these genomic resources, the establishment of a standardized reverse transcriptase-quantitative polymerase chain reaction (RT-qPCR) procedure in *F*. *occidentalis* according to the MIQE (Minimum Information for publication of Quantitative real-time PCR Experiments) guidelines is instrumental for functional genomics studies of this pest [13].

RT-qPCR is a high-throughput technique for measuring and evaluating gene expression [14]; however, there remain limitations that can significantly influence the normalization of gene expression, including sample amount, RNA quality and quantity, efficiency of reverse transcription, cDNA quality, PCR efficiency, and experimental operation between different samples [15, 16]. Previously, *Actin* was used as stable reference gene to investigate the TSWV titers in *F*. *occidentalis* [12, 17–19], whereas another reference gene, *RP49*, was used as an internal control gene to estimate the abundance of TSWV N RNA in *F*. *occidentalis* [10]. The *18S* gene was used as an endogenous control to investigate the expression profiles of 36 selected genes in viruliferous and non-viruliferous *F*. *occidentalis* [8], however, only one reference gene for the RT-qPCR experiment was used in these studies [8–11, 17–19]. In standardized RT-qPCR experiments, two or several reference genes are commonly used to normalize gene expression data, which are stably expressed across various experimental conditions and serve as the internal controls [14–16]. Recently, seven candidate genes from *F*. *occidentalis* including *Actin*, *18S*, *H3*, *Tubulin*, *GAPDH*, *EF*-*1A*, and *RPL32* were evaluated for their suitability as reference genes across different developmental stages and temperatures [20]. A previous study strongly suggests the necessity of conducting custom reference gene selection designed for all experimental conditions, even when examining the same abiotic or biotic factor [21].

The objective of this study was to determine most stable reference genes in nonviruliferous and viruliferous *F*. *occidentalis*. Here, 11 candidate reference genes from *F*. *occidentalis* were tested, namely, *β-actin* (*Actin*), *α-tubulin* (*Tubulin*), *elongation factor 1 α* (*EF1A*), *vacuolar-type H* ^+^ *-ATPase* (*ATPase*), *NADH-ubiquinone oxidoreductase* (*NADH*), *heat shock protein 60* (*HSP60*), *heat shock protein 70* (*HSP70*), *heat shock protein 90* (*HSP90*), *ribosomal protein l32* (*RPL32*), *28S ribosomal RNA* (*28S*), and *18S ribosomal RNA* (*18S*). To validate the selected reference genes, these candidates were further examined by RT-qPCR analysis against a TSWV-receptor gene in both nonviruliferous and viruliferous *F*. *occidentalis*.

## Materials and Methods

### Ethics Statement

The western flower thrips *F*. *occidentalis* (Pergande) (Thysanoptera: Thripidae) was collected from clover plants, *Trifolium repens* L., at the Experimental Station of Qingdao Agricultural University. No specific permit was required for the collection described.

### Insect rearing, plant cultures, and TSWV inoculation

*F*. *occidentalis* was reared on the common bean *Phaseolus vulgaris* and maintained in MGC-250BP-2 incubators (Shanghai Yiheng Instruments, China) at 55–60% relative humidity and under a light: dark cycle of 16: 8 h. *F*. *occidentalis* adults were allowed to lay eggs on *P*. *vulgaris* for 1 day, and the adults were subsequently removed. First instar larvae were obtained in a few days.

Pepper (*Capsicum annuum* L., cv. Zhongjiao 6) plants were grown in soil mixed with vermiculite and organic fertilizer in 1.5-L pots (one plant per pot) under natural light and controlled temperatures (30 ± 2°C) in a greenhouse.

TSWV was maintained on *Datura stramonium* L. (Solanaceae). TSWV-infected pepper plants were prepared according to the method described in our previous study [22]. Control plants were treated similarly, but were inoculated by applying ground healthy plant material.

### Nonviruliferous and viruliferous *F*. *occidentalis*

Healthy and TSWV-infected pepper leaf discs (diameter, 26 mm) were obtained using a cork borer. Each leaf was kept in a ventilated vial containing 3 ml of 1.0% agar to keep them fresh. Thirty newly hatched first instar larvae (nymphs) as one replicate were maintained on healthy or TSWV-infected discs in respective vials for 24 h to obtain nonviruliferous and viruliferous *F*. *occidentalis*, respectively [23]. Each treatment consisted of four replicates. Samples were frozen in liquid nitrogen and stored at –80°C.

### Total RNA extraction and cDNA synthesis

Total RNA was extracted using TRIzol reagent (Invitrogen, Carlsbad, CA) according to previously described methods [24]. First-strand cDNA was synthesized from 1.0 μg of total RNA using a PrimeScript RT Reagent Kit with gDNA Eraser (TaKaRa, Shiga, Japan) according to the manufacturer’s recommendations. cDNA was diluted 10-fold for subsequent RT-qPCR studies.

### Reference gene selection and primer design

Eleven candidate reference genes were selected that are commonly used in RT-qPCR and that have been verified as stable genes in other species (Table 1). Primers were designed based on the sequences obtained from GenBank (Table 1). The primers used for RT-qPCR analysis were designed online (https://www.idtdna.com/Primerquest/Home/Index) using the following parameters: amplicon length 75–150 bp with the optimum amplicon at 100 bp, Tm 59–65°C with the optimum amplicon at 62°C, primer lengths 17–30 bp, optimized to 22 bp, and GC content 35–65% with the optimal content at 50%. PCR amplifications were performed in 25-μl reactions containing 2.5 μl of 10× PCR Buffer (Mg^2+^ Plus), 0.5 μl of dNTP mix (10 mM of each nucleotide), 0.5 μl of each primer (10 μM each), and 0.25 μl of TaKaRa Taq (5 U/μl) (TaKaRa). Target genes were amplified using the following parameters: initial denaturation at 94°C for 3 min; 35 cycles of 94°C for 30 s, 59°C for 45 s, and 72°C for 1 min; and a final elongation step of 72°C for 10 min. Primer specificity was confirmed by melting curve analysis and agarose gel electrophoresis of the amplification product.

**Table 1:** Summary of the 11 housekeeping genes tested in this study.

Gene	Description	Accession No.	Primer sequences (5’-3’)	Length (bp)	E(%)*	R^2,^ **
*HSP70*	*heat shock protein 70*	KC148536	F: GTCACCGTACCCGCATATTT	104	0.95	0.9845
			R: GCAGTGGGCTCGTTGATAATA			
*HSP60*	*heat shock protein 60*	JX967580	F: CTGGACTGTAAGCGTGCTATAA	80	0.91	0.9903
			R: GGCACGATGAACACCTATGA			
*EF1A*	*elongation factor 1 α*	AB277244	F: AAGGAACTGCGTCGTGGATA	99	1.05	0.991
			R: AGGGTGGTTCAGGACAATGA			
*RPL32*	*ribosomal protein l32*	AB572580	F: CTGGCGTAAACCTAAGGGTATT	96	0.98	0.9998
			R: GTCTTGGCATTGCTTCCATAAC			
*ATPase*	*vacuolar type H* ^+^ *-ATPase*	JN835456	F: TACCAAATGGGACTCCAATACC	130	0.90	0.9970
			R:GTAAGTAAGAGGTGGCCAGATAC			
*HSP90*	*heat shock protein 90*	JX967579	F: CTCGCAACCAGGACGATATTAG	110	0.96	0.9918
			R: CTGACCCTCCACAGAGAAATG			
*NADH*	*NADH-ubiquinone oxidoreductase*	YP_006576366	F: AGCTACTAAACCGCCTCATAAA	99	0.95	0.9656
			R:GGTGGTTATGGTATTTATCGTTTGT			
*18S*	*18S ribosomal RNA*	JX002704	F: CTGCGGAAATACTGGAGCTAATA	109	1.09	0.9960
			R: AAGTAGACGATGGCCGAAAC			
*Actin*	*β-actin*	AF434716	F:CCTCATCCCTAGTTGTCTTGTG	96	0.86	0.9788
			R: TTCTCGCTCAGCTGTAATTGT			
*28S*	*28S ribosomal RNA*	GU980314	F: GGGTGGTAAACTCCATCTAAGG	108	0.97	0.9969
			R:CACGTACTCTTGAACTCTCTCTTC			
*Tubulin*	*α-tubulin*	KC513334	F: GTGGACAACGAAGCCATCTA	77	1.04	0.9900
			R: CGGTTCAGGTTGGTGTAGG			

"*": PCR efficiency (calculated from the standard curve)

"**": Regression coefficient

### RT-qPCR

RT-qPCR was performed on a qTOWER 2.2 Real-Time Thermal Cycler system (Analytik Jena, Germany). PCR reactions (20 μl) contained 7.2 μl of ddH_2_O, 10.0 μl of 2× SYBR Premix Ex Taq (TaKaRa), 0.4 μl of each specific primer (10 μM), and 2.0 μl of first-strand cDNA. The RT-qPCR program amplified target genes using the following parameters: initial denaturation for 3 min at 95°C followed by 40 cycles of denaturation at 95°C for 15 s and annealing at 60°C for 30 s. For melting curve analysis, a dissociation step cycle (55°C for 10 s, and then an increase of 0.5°C every 10 s up to 95°C) was used. The reactions were set up in 96-well Microseal PCR plates (Sangon, Shanghai, China) in triplicate. A 5-fold dilution series of cDNA (1/5, 1/25, 1/125, 1/625, and 1/3125) was used to construct a standard curve. The RT-qPCR efficiency was calculated according to the following equation: E = (10^[-1/slope]^ -1)×100.

### Validation of selected reference genes

One TSWV-receptor gene of *F*. *occidentalis* was used to evaluate the candidate reference genes (GenBank No. AF247969). TSWV-receptor gene expression levels were investigated in nonviruliferous and viruliferous *F*. *occidentalis*. Two normalization factors (NFs) were calculated based on (1) the geometric mean of genes with the lowest *Geomean* values, and (2) a single reference with the lowest or highest *Geomean* value (as determined by *RefFinder*). Relative quantification of the TSWV-receptor gene in different samples was performed using the 2^*-ΔΔCt*^ method [25].

### Data analysis

All biological replicates were used to calculate the average *C*_*t*_ value. The stability of candidate reference genes was evaluated by the algorithms *geNorm* [14], *NormFinder* [15], *BestKeeper* [26], and the *ΔC*_*t*_ method [27]. Finally, the tested candidates were compared and ranked using the web-based comprehensive analysis tool *RefFinder* (http://www.leonxie.com/referencegene.php). Nonparametric tests (K independent samples) were used to compare the expression levels of the TSWV-receptor gene in viruliferous and nonviruliferous *F*. *occidentalis*. Statistical analysis was conducted using SPSS 20.0 (SPSS Inc., Chicago, IL, USA).

## Results

### Transcriptional profiling of candidate reference genes

All tested genes were visualized as a single amplicon of the expected size on a 2.0% agarose gel (S1 Fig). Furthermore, gene-specific amplification was confirmed by a single peak in real-time melting curve analysis (S2 Fig). The linear regression equation, correlation coefficient, and PCR efficiency for each standard curve are shown in Table 1.

**Fig 1:**
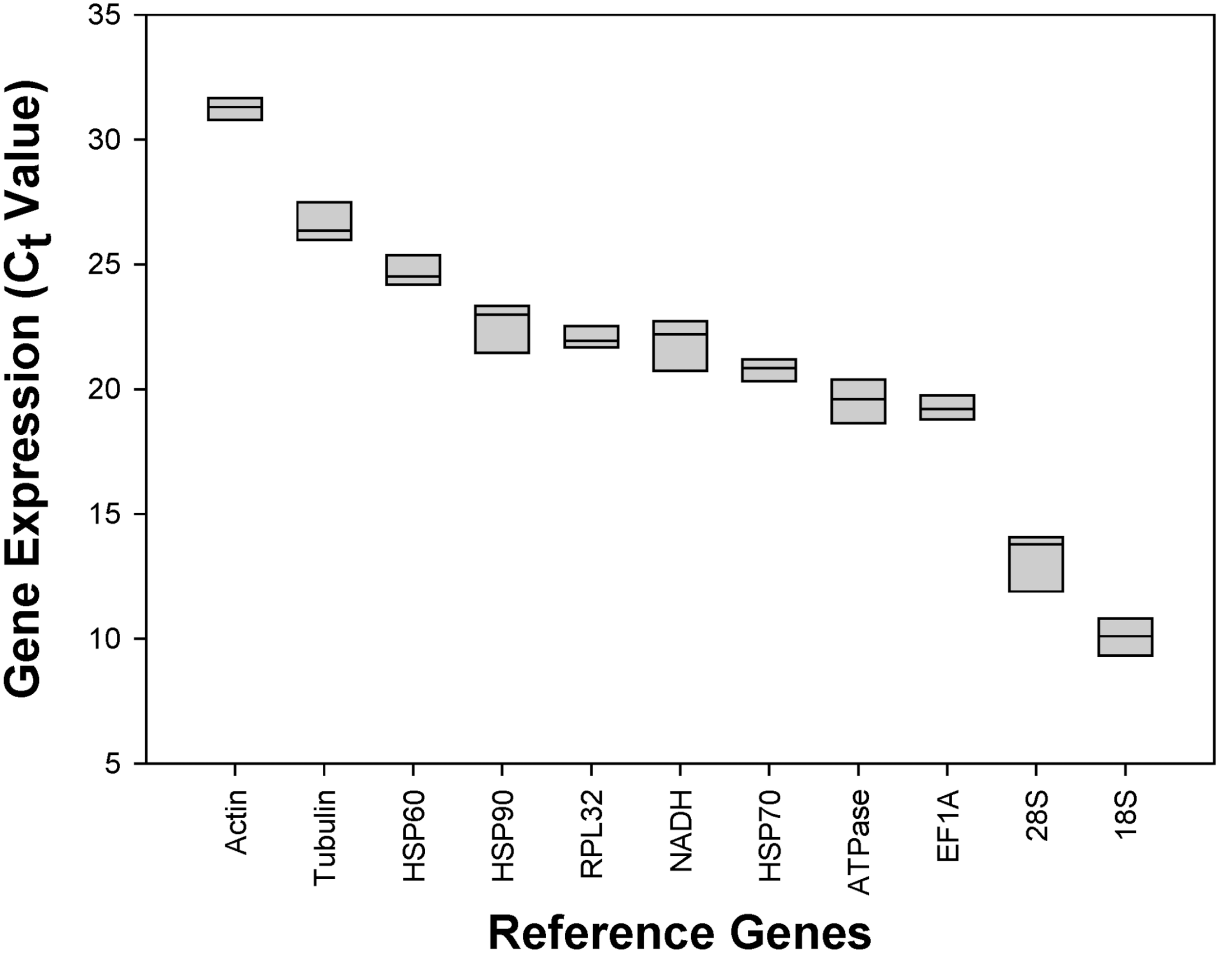
Expression profiles of 11 candidate reference genes in *Frankliniella occidentalis*. The expression levels of the candidate reference genes are documented by the *C*_*t*_ values. The median is represented by the line in the box. The interquartile rang is bordered by the upper and lower edges, which indicate the 75^th^ and 25^th^ percentiles, respectively.

The mean and standard deviation (SD) of *C*_*t*_ values were calculated for all samples (S1 Table). *EF1A* (SD = 0.68) had the least variable expression and this was reflected in its low SD values. By contrast, *28S* (SD = 1.35) had the most variable expression, as shown by its high SD values. Additionally, *18S* had the lowest *C*_*t*_ values (*C*_*tavg*_ = 10.09), suggesting that it had the highest expression level, whereas *Actin* was the lowest expressed gene among the candidates (*C*_*tavg*_ = 31.62) (Fig 1; S1 Table).

### Stability of candidate reference genes

*GeNorm* determines its ranking based on the geometric mean of the SD of each transformed gene pair combination (M-value). The lower the M-value, the higher the ranking. *EF1A* and *HSP70* were co-ranked as the most stable genes (M = 0.270). The overall order based on *geNorm* from the most stable to the least stable reference gene was as follows: *EF1A* = *HSP70*, *RPL32*, *HSP60*, *ATPase*, *HSP90*, *NADH*, *28S*, *18S*, *Actin*, and *Tubulin* (Table 2).

**Table 2:** Ranking of the 11 housekeeping genes using five different algorisms.

*RefFinder*		*geNorm*		*NormFider*		*ΔC*_*t*_		*BestKeeper*	
Genes	GM	Genes	SV	Genes	SV	Genes	SV	Genes	SD
*HSP70*	1.68	*EF1A*	0.270	*HSP60*	0.152	*HSP60*	0.69	*EF1A*	0.499
*HSP60*	2.00	*HSP70*	0.270	*HSP70*	0.299	*HSP70*	0.71	*HSP70*	0.521
*EF1A*	2.11	*RPL32*	0.335	*RPL32*	0.398	*RPL32*	0.75	*RPL32*	0.556
*RPL32*	3.00	*HSP60*	0.375	*EF1A*	0.507	*HSP90*	0.80	*HSP60*	0.560
*ATPase*	5.89	*ATPase*	0.537	*ATPase*	0.531	*EF1A*	0.80	*Actin*	0.566
*HSP90*	6.16	*HSP90*	0.592	*HSP90*	0.547	*ATPase*	0.82	*18S*	0.595
*NADH*	7.45	*NADH*	0.627	*NADH*	0.638	*NADH*	0.85	*Tubulin*	0.784
*18S*	8.13	*28S*	0.667	*28S*	0.852	*28S*	1.00	*ATPase*	0.809
*Actin*	8.41	*18S*	0.754	*18S*	0.878	*18S*	1.06	*NADH*	0.894
*28S*	8.66	*Actin*	0.815	*Actin*	0.893	*Actin*	1.08	*HSP90*	0.915
*Tubulin*	9.82	*Tubulin*	0.888	*Tubulin*	1.060	*Tubulin*	1.22	*28S*	1.069

 The *ΔC*_*t*_ method relies on relative pair-wise comparisons. Using raw *C*_*t*_ values, the average SD of each gene set is inversely proportional to its stability. *HSP60* (0.69) was the top-ranked gene (Table 2). The overall order from the most stable to the least stable reference gene based on the *ΔC*_*t*_ method was as follows: *HSP60*, *HSP70*, *RPL32*, *HSP90*, *EF1A*, *ATPase*, *NADH*, *28S*, *18S*, *Actin*, and *Tubulin* (Table 2; S2 Table).

A low stability value suggests a more stable gene by *NormFinder*. *HSP60* (0.152) was the most reliable and stable reference gene. The overall order from the most stable to the least stable reference gene based on *NormFinder* was as follows: *HSP60*, *HSP70*, *RPL32*, *EF1A*, *ATPase*, *HSP90*, *NADH*, *28S*, *18S*, *Actin*, and *Tubulin* (Table 2).

A low SD value suggests a more stable gene by *BestKeeper*. The stability of a gene is inversely proportional to the SD value. *EF1A* (SD = 0.499) had the least variable expression levels across all samples (Table 2). The overall order from the most stable to the least stable reference gene based on *BestKeeper* was as follows: *EF1A*, *HSP70*, *RPL32*, *HSP60*, *Actin*, *18S*, *Tubulin*, *ATPase*, *NADH*, *HSP90*, and *28S* (Table 2).

### Quantitative analysis of candidate reference genes based on *geNorm*

**Fig 2:**
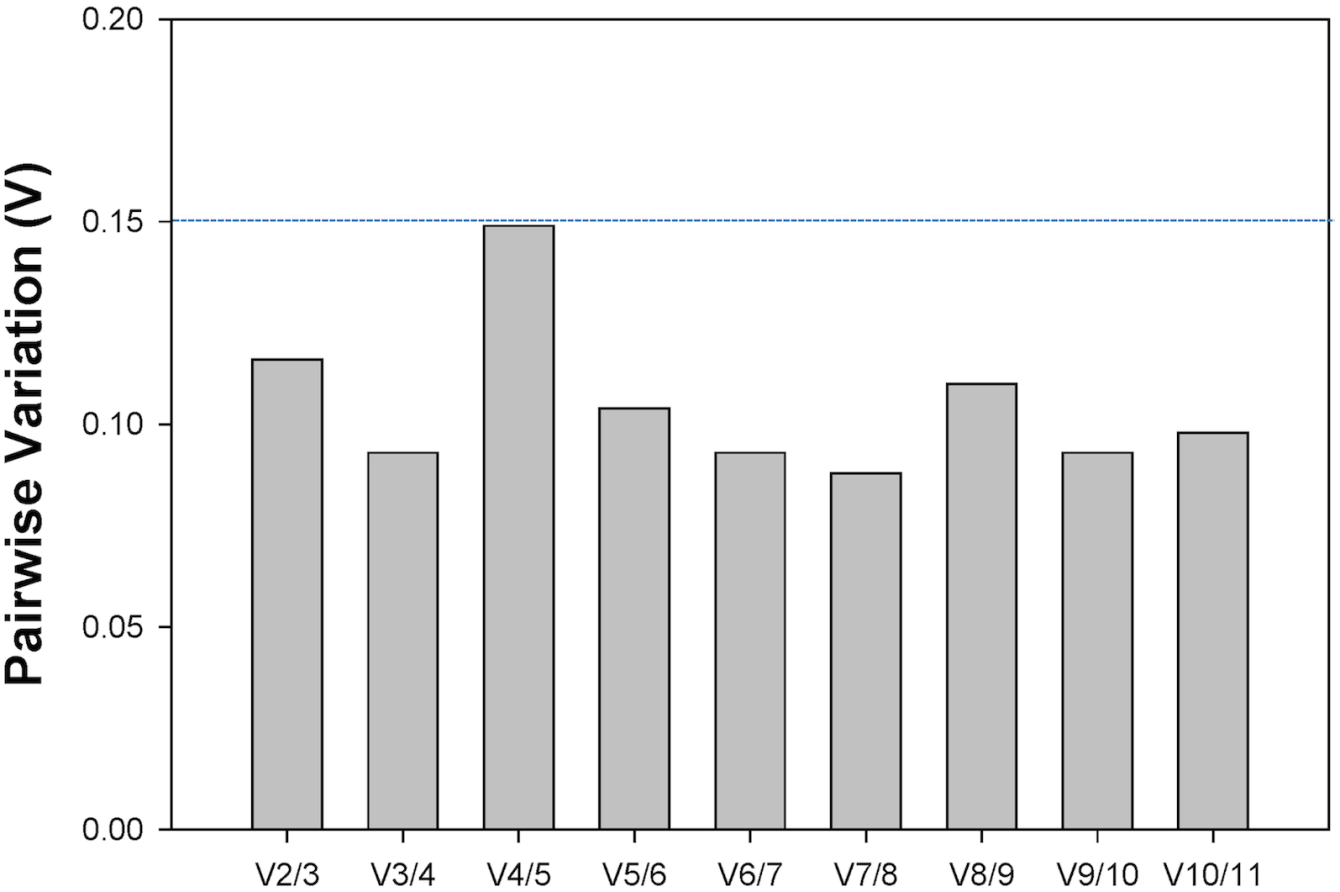
Determination of the optimal number of reference genes under plant virus stress. To determine the minimum number of genes required for normalization, the V-value was computed using *geNorm*. Starting with two genes, the software sequentially adds another gene and recalculates the NF ratio. If the added gene does not increase the NF ratio above the proposed 0.15 cut-off value, then the starting pair of genes is sufficient for normalization. If the NF ratio is adequately increased, more genes should be incorporated.

Under plant virus stress, the first V-value less than 0.15 was after V2/3 (Fig 2). This means that two reference genes were sufficient for reliable normalization regardless of the virus infection status of the insect.

### Comprehensive ranking of the best reference genes using *RefFinder*

According to *RefFinder*, which integrates the above-mentioned four software tools to compare and rank candidates, the comprehensive ranking of candidate reference genes from the most to the least stable was as follows: *HSP70*, *HSP60*, *EF1A*, *RPL32*, *ATPase*, *HSP90*, *NADH*, *18S*, *Actin*, *28S*, and *Tubulin* (Table 2). Among these, *Tubulin* had a geometric mean of almost 10.0, and was determined to be the least suitable candidate to serve as a reliable reference gene for normalizing gene expression.

### Validation of selected reference genes

**Fig 3:**
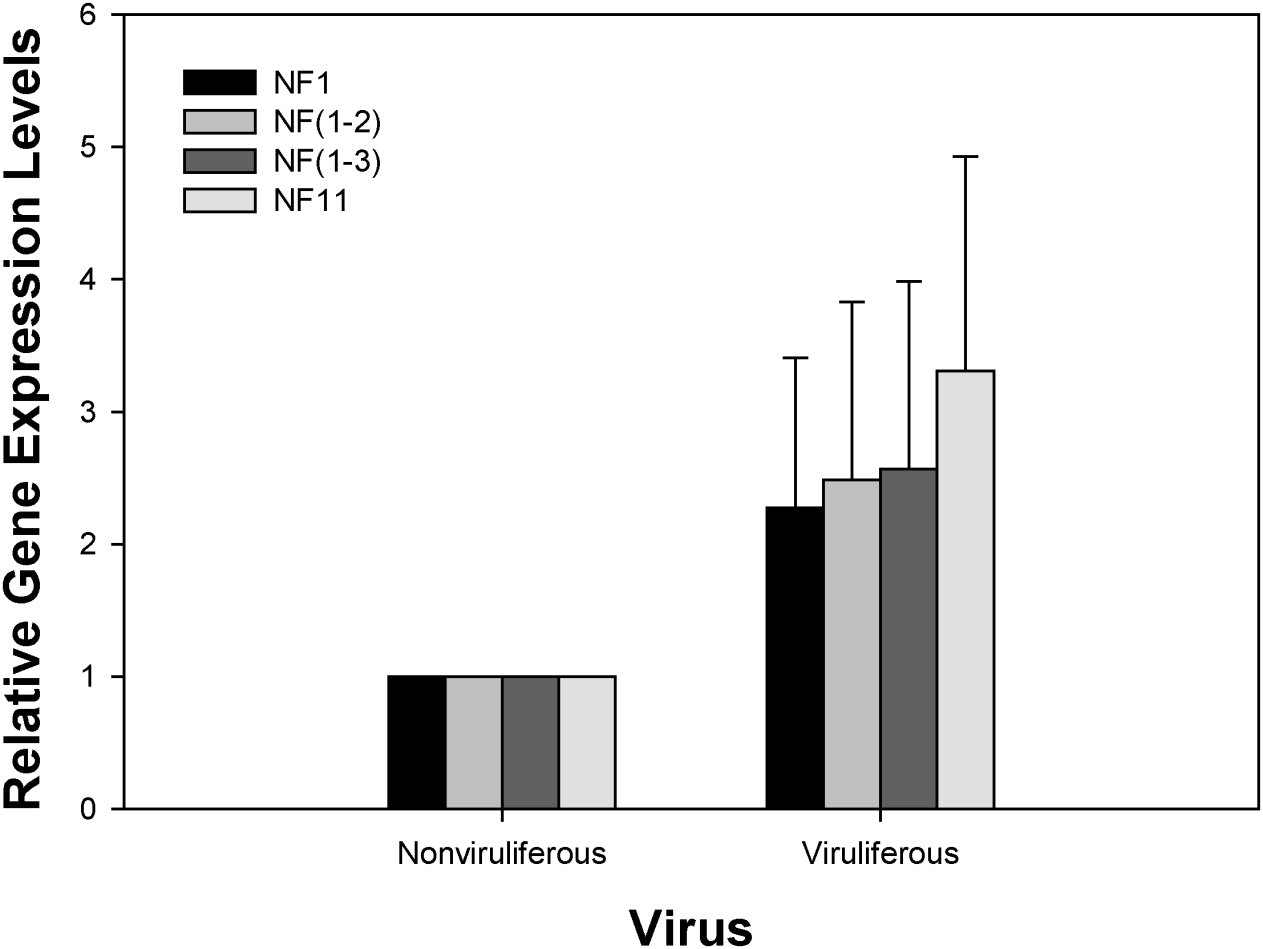
Validation of the recommended reference gene. The expression profiles of the TSWV-receptor gene in nonviruliferous and viruliferous *Frankliniella occidentalis* were investigated using different combinations of reference genes. NF1, NF (1–2), NF (1–3), and NF9 indicate that expression of the TSWV-receptor gene was normalized using the best, top two, top three, and worst reference genes, respectively. The bar represents the mean and standard error of four biological replicates.

Using one, two, or three of the best reference gene combinations for normalization, expression of the TSWV-receptor gene did not differ between viruliferous and nonviruliferous *F*. *occidentalis* (*P* > 0.05) (Fig 3).

## Discussion

Recent studies suggest that there is no single reference gene that is suitable for all types of normalization experiments [14, 28–30]. Therefore, candidate reference genes should be evaluated before they are used for normalization [21, 31]. Recently, a study found that the ranking of candidate reference genes in the sweet potato whitefly, *Bemisia tabaci* varied substantially among intra- and inter-classes of insecticides [21]. This strongly suggested the need for conducting reference gene selection specifically designed for all experimental conditions, even when examining the same abiotic or biotic factor [21]. Zheng et al. (2014) focused on the selection of reference genes across different developmental stages and temperatures in *F*. *occidentalis* [20], while this study mainly focused on the impact of virus infection on the stability of these internal controls. As expected, these selected reference genes varied across different experimental conditions. For example, our study demonstrated that *Tubulin* was the least appropriate reference gene in *F*. *occidentalis*, which is inconsistent with Zheng et al. (2014) where *Tubulin* was a suitable reference gene for *F*. *occidentalis* across different experimental conditions [20]. In addition, *Actin* was not appropriate in our study, which was consistent with the Zheng et al. (2014) study [20]. Interestingly, *Actin* has been used previously as reference gene to investigate the TSWV titer in *F*. *occidentalis* [12, 17–19]. Therefore, we suggest that custom reference gene selection should be conducted for each experimental condition.

In previous studies, only one reference gene was selected for the RT-qPCR experiment in *F*. *occidentalis* [8–11, 17–19]. To avoid biased normalization, many researchers have started to advocate the use of multiple reference genes to analyze gene expression [14, 24, 28–30]. The *geNorm* program first calculates an expression stability value (M) for each gene and compares the pair-wise variation (V) of this gene with the other genes. A threshold of V<0.15 was suggested for valid normalization. *geNorm* starts with a single gene pair, and tests whether the inclusion of a 3^rd^ gene adds significant variation. The pair-wise variation (V_n_/V_n+1_) was analyzed between the normalization factors NF_n_ and NF_n+1_ by *geNorm* to determine the optimal number of references genes required for qRT-PCR data normalization. In our study, the first pair-wise variation value less than 0.15 was after V2/3 (Fig 2), suggesting that two reference genes are sufficient for studying gene expression in nonviruliferous and viruliferous *F*. *occidentalis* (Fig 2).

In short, 11 candidate reference genes were selected for RT-qPCR analysis in nonviruliferous and viruliferous *F*. *occidentalis* assessed by five algorithms (*geNorm*, *NormFinder*, *BestKeeper*, *ΔC*_*t*_ method, and *RefFinder*). Among them, *HSP60*, *HSP70*, and *RPL32* are the three most stable reference genes under the impact of plant virus infection as found by all five algorithms. Recently, the introduction of RNAi as a tool for functional genomics assays for *F*. *occidentalis* was developed [28]. Therefore, our study not only provides a standardized protocol for the quantification of gene expression in *F*. *occidentalis*, but also provides a solid foundation for genomic and functional genomics research assessing the interactions between TSWV and *F*. *occidentalis*.

## Supporting Information

S1 Fig The agrose gel electrophoresis of the eleven candidate reference genes. M,DL 2000 bp Marker; Templates in the PCR reactions were as follows: 1) *18S*; 2) *28S*; 3) *Actin*; 4) *ATPase*; 5) *EF1A*; 6) *HSP60*; 7) *HSP70*; 8) *HSP90*; 9) *NADH*; 10) *RPL32*; 11) *Tubulin*.
(TIF)

S2 Fig Melting curves of eleven candidate reference genes in *Frankliniella occidentalis*. (TIF)

S1 Table The mean and standard deviation (SD) of the *C*_*t*_ value for each candidate reference gene. (DOCX)

S2 Table Summary of mean and SD values of gene pairwise comparison using the *ΔC*_*t*_ method. (DOCX)
