# Supplementary figures and images for: Stable Reference Gene Selection for RT-qPCR Analysis in Nonviruliferous and Viruliferous Frankliniella occidentalis
Source: PLoS One. 2015 Aug 5;10(8):e0135207. doi: 10.1371/journal.pone.0135207 (PMC4526564; doi:10.1371/journal.pone.0135207)

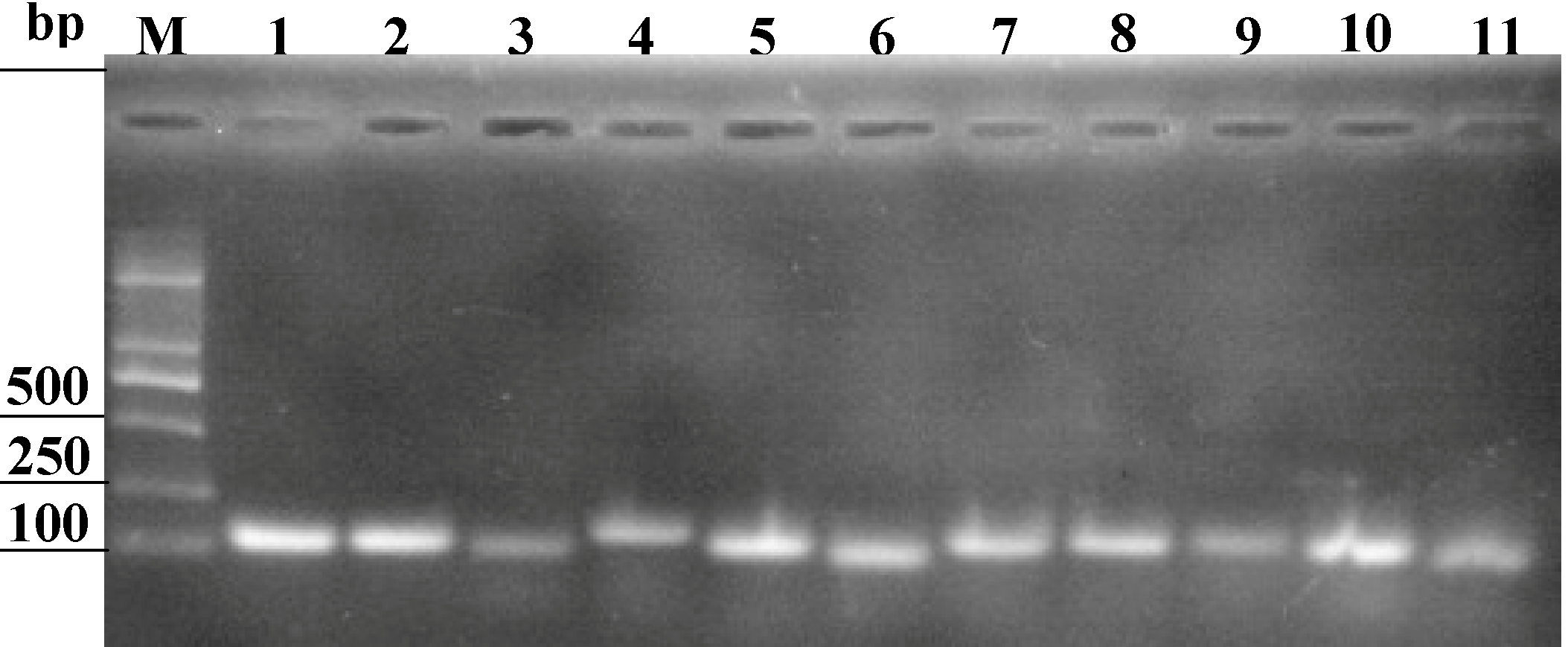

Supplement: S1 Fig — M,DL 2000 bp Marker; Templates in the PCR reactions were as follows: 1) 18S; 2) 28S; 3) Actin; 4) ATPase; 5) EF1A; 6) HSP60; 7) HSP70; 8) HSP90; 9) NADH; 10) RPL32; 11) Tubulin. (TIF) [file pone.0135207.s001.tif]

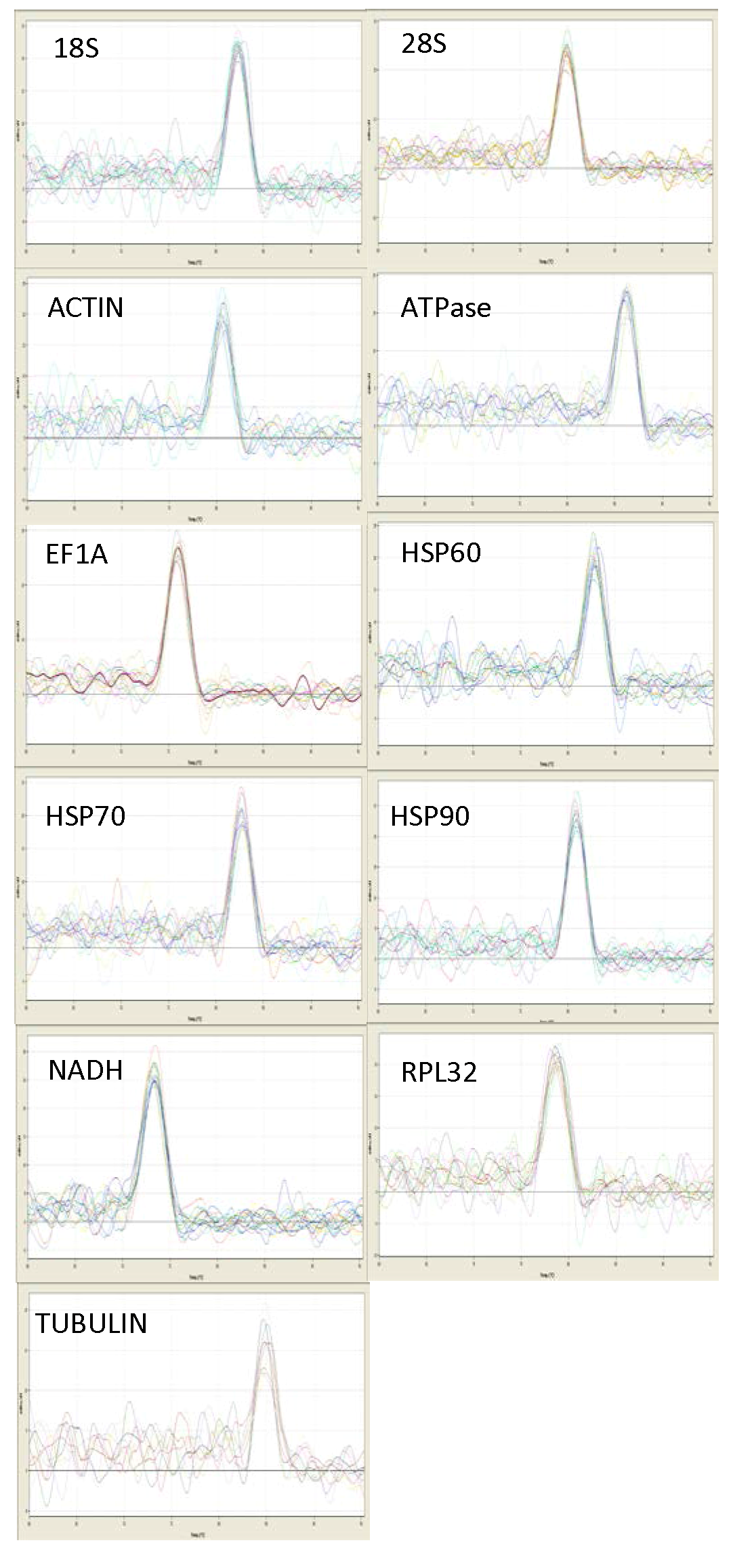

Supplement: S2 Fig — (TIF) [file pone.0135207.s002.tif]
