# Supplementary material for: Stable Reference Gene Selection for RT-qPCR Analysis in Nonviruliferous and Viruliferous Frankliniella occidentalis
Source: PLoS One. 2015 Aug 5;10(8):e0135207. doi: 10.1371/journal.pone.0135207 (PMC4526564; doi:10.1371/journal.pone.0135207)
Supplement: S1 Table — (DOCX) [file pone.0135207.s003.docx]

**Table S1. The mean and standard deviation (SD) of the *C_t_* value for each candidate reference gene**

| Genes | *C_t_* ±SD |
| --- | --- |
| *Actin* | 31.26±0.84 |
| *Tubulin* | 26.72±0.94 |
| *HSP60* | 24.63±0.72 |
| *HSP90* | 22.55±1.08 |
| *RPL32* | 22.11±0.78 |
| *NADH* | 21.89±1.11 |
| *HSP70* | 20.82±0.65 |
| *ATPase* | 19.59±0.97 |
| *EF1A* | 19.34±0.68 |
| *28S* | 13.16±1.35 |
| *18S* | 10.09±0.73 |
