# Supplementary material for: Stable Reference Gene Selection for RT-qPCR Analysis in Nonviruliferous and Viruliferous Frankliniella occidentalis
Source: PLoS One. 2015 Aug 5;10(8):e0135207. doi: 10.1371/journal.pone.0135207 (PMC4526564; doi:10.1371/journal.pone.0135207)
Supplement: S2 Table — (DOCX) [file pone.0135207.s004.docx]

**Table S2. Summary of mean and SD values of gene pairwise comparison using the *ΔC_t_* method**

| *Gene* |  | Pair 1 | Pair 2 | Pair 3 | Pair 4 | Pair 5 | Pair 6 | Pair 7 | Pair 8 | Pair 9 | Pair 10 | Average SD |
| --- | --- | --- | --- | --- | --- | --- | --- | --- | --- | --- | --- | --- |
| *18S* | Mean | 3.08 | 21.17 | 9.51 | 9.25 | 14.55 | 10.73 | 12.46 | 11.81 | 12.02 | 16.63 |  |
|  | SD | 1.55 | 0.91 | 1.11 | 0.69 | 0.91 | 0.77 | 1.26 | 1.35 | 0.82 | 1.18 | 1.06 |
| *28S* | Mean | -3.08 | 18.10 | 6.43 | 6.18 | 11.47 | 7.66 | 9.38 | 8.73 | 8.95 | 13.56 |  |
|  | SD | 1.55 | 1.47 | 0.69 | 1.14 | 0.88 | 0.96 | 0.45 | 0.41 | 0.99 | 1.44 | 1.00 |
| *Actin* | Mean | -21.17 | -18.10 | -11.67 | -11.92 | -6.63 | -10.44 | -8.72 | -9.37 | -9.15 | -4.54 |  |
|  | SD | 0.91 | 1.47 | 0.99 | 1.06 | 0.81 | 0.98 | 1.19 | 1.20 | 0.88 | 1.32 | 1.08 |
| *ATPase* | Mean | -9.51 | -6.43 | 11.67 | -0.26 | 5.04 | 1.23 | 2.95 | 2.30 | 2.52 | 7.13 |  |
|  | SD | 1.11 | 0.69 | 0.99 | 0.88 | 0.69 | 0.78 | 0.39 | 0.48 | 0.77 | 1.39 | 0.82 |
| *EF1A* | Mean | -9.25 | -6.18 | 11.92 | 0.26 | 5.30 | 1.48 | 3.21 | 2.56 | 2.77 | 7.38 |  |
|  | SD | 0.69 | 1.14 | 1.06 | 0.88 | 0.53 | 0.27 | 0.89 | 1.04 | 0.43 | 1.08 | 0.80 |
| *HSP60* | Mean | -14.55 | -11.47 | 6.63 | -5.04 | -5.30 | -3.82 | -2.09 | -2.74 | -2.53 | 2.09 |  |
|  | SD | 0.91 | 0.88 | 0.81 | 0.69 | 0.53 | 0.35 | 0.66 | 0.72 | 0.37 | 0.96 | 0.69 |
| *HSP70* | Mean | -10.73 | -7.66 | 10.44 | -1.23 | -1.48 | 3.82 | 1.73 | 1.07 | 1.29 | 5.90 |  |
|  | SD | 0.77 | 0.96 | 0.98 | 0.78 | 0.27 | 0.35 | 0.76 | 0.87 | 0.31 | 1.01 | 0.71 |
| *HSP90* | Mean | -12.46 | -9.38 | 8.72 | -2.95 | -3.21 | 2.09 | -1.73 | -0.65 | -0.44 | 4.18 |  |
|  | SD | 1.26 | 0.45 | 1.19 | 0.39 | 0.89 | 0.66 | 0.76 | 0.23 | 0.82 | 1.32 | 0.80 |
| *NADH* | Mean | -11.81 | -8.73 | 9.37 | -2.30 | -2.56 | 2.74 | -1.07 | 0.65 | 0.22 | 4.83 |  |
|  | SD | 1.35 | 0.41 | 1.20 | 0.48 | 1.04 | 0.72 | 0.87 | 0.23 | 0.93 | 1.28 | 0.85 |
| *RPL32* | Mean | -12.02 | -8.95 | 9.15 | -2.52 | -2.77 | 2.53 | -1.29 | 0.44 | -0.22 | 4.61 |  |
|  | SD | 0.82 | 0.99 | 0.88 | 0.77 | 0.43 | 0.37 | 0.31 | 0.82 | 0.93 | 1.17 | 0.75 |
| *Tubulin* | Mean | -16.63 | -13.56 | 4.54 | -7.13 | -7.38 | -2.09 | -5.90 | -4.18 | -4.83 | -4.61 |  |
|  | SD | 1.18 | 1.44 | 1.32 | 1.39 | 1.08 | 0.96 | 1.01 | 1.32 | 1.28 | 1.17 | 1.22 |
